# PulseNet Latin America and the Caribbean Network: Present and Future

**DOI:** 10.1089/fpd.2018.2587

**Published:** 2019-07-09

**Authors:** Isabel Chinen, Josefina Campos, Tshewang Dorji, Enrique Pérez Gutiérrez

**Affiliations:** ^1^ National Administration of Laboratories and Health Institutes - ANLIS “Dr. Carlos G. Malbrán,” Buenos Aires, Argentina.; ^2^ Health Emergency Information and Risk Assessment, Health Emergencies Department, Pan American Health Organization/World Health Organization (PAHO/WHO), Washington, District of Columbia.

**Keywords:** surveillance, Public Health, Foodborne Pathogens and Disease Surveillance

## Abstract

PulseNet Latin America and Caribbean (PNLAC) was established in 2003 and is one of seven Regional networks within PulseNet International. The main objectives of the network are to strengthen national and regional laboratory-based foodborne disease surveillance for early detection and investigation of outbreaks to setup control and prevention strategies in contribution to Public Health. Participants perform standardized pulsed-field gel electrophoresis (PFGE) protocols and analysis. For functioning, it is important for the network, the development of national and regional databases (RDBs) and the communication between countries, regionally and internationally. Metadata from over 8600 cases/outbreaks are profiled and isolated by PFGE and are incorporated into the RDB, hosted by the Pan American Health Organization. Currently PNLAC is moving toward whole-genome sequencing to use as a complementary strategy for surveillance. The aim of this article was to describe the experience of the construction of PNLAC, and its contribution to the surveillance of the foodborne diseases at the country and regional levels.

## Introduction

PulseNet Latin America and Caribbean (PNLAC) was established in 2003 and is one of seven regional networks within PulseNet International (PNInt). The main objective is to strengthen national and regional laboratory-based foodborne disease surveillance for early detection and investigation of outbreaks, to setup control, and prevention strategies in contribution to Public Health. Participants perform standardized pulsed-field gel electrophoresis (PFGE) protocols and analysis. Each country creates the national database (NDB) with PFGE patterns obtained using standardized protocols to monitor the strains circulating in the country according to how each country established their surveillance system, and works in connection with other participants through the regional database (RDB), in contribution to the regional surveillance. Metadata from over 8600 cases/outbreaks are profiled and isolated by PFGE and are incorporated into the RDB, hosted by the Pan American Health Organization (PAHO). Currently, PNLAC is moving toward whole-genome sequencing (WGS) to use as a complementary strategy for surveillance.

Participants of PNLAC are considered low-income countries, regarding their social and economic contexts. Nevertheless, we have recognized the need of the integration as region into the global surveillance. Therefore, the building of the network was accomplished with the effort of each PNLAC member, with a process of training in subtyping techniques, and also learning how to work as a team. The continued growth was essential to maintain the results comparable to international standards. It is important to mention that the functioning was partially supported by different international partnerships. The aim of this article is to describe the experience of the construction of PNLAC network, and its contribution to foodborne disease surveillance at the country and regional levels.

## New Network Structure

**FIG. 1.**
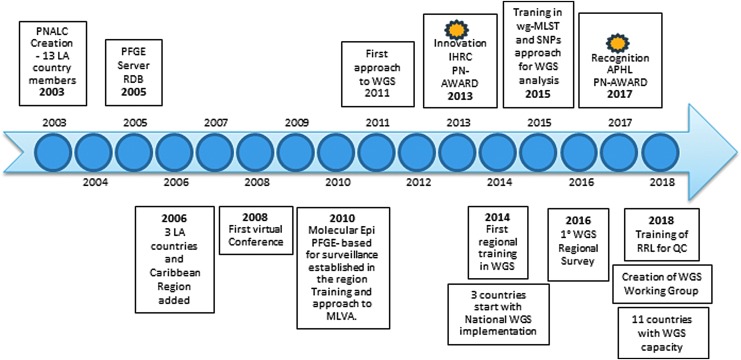
Timeline marking the progress of PNLAC. Main events important to the enhancement of the network. APHL, American Public Health Laboratories; IHRC, International Consulting; LA, Latin America; MLVA, Multiple-Locus Variable-Number Tandem Repeat Analysis; PFGE, pulsed-field gel electrophoresis; PN, PulseNet; QC, quality control; RDB, regional database; RRL, Regional Reference Laboratory; wg-MLST, whole genome-Multilocus Sequence Typing; WGS, whole genome sequencing.

PNLAC has grown since its creation in 2003 when it started with 13 founding members. Between 2006 and 2013, PNLAC included three additional countries (Ecuador, Guatemala, Panamá) and the 22 countries of the Caribbean Region through the integration of Caribbean Public Health Agency (CARPHA) Subregional reference laboratory (RRL), into the network. Since 2014, food and animal agency laboratories in Mexico, Colombia, and Argentina joined PNLAC as part of the National Surveillance. Today, PNLAC network is made up of 24 reference laboratories/agencies from 16 countries of Latin America (LA) and the Caribbean Region: Argentina, Bolivia, Brazil, Chile, Colombia, Costa Rica, Cuba, Ecuador, Guatemala, Nicaragua, México, Panamá, Paraguay, Perú, Uruguay, Venezuela, and CARPHA. Each member country is part of the assembly that represents the highest authority in the network within the boundaries of its expertise. The members are in charge of the implementation of the subtyping techniques, and fulfill the requirement to work and exchange information in the network. Technical Coordination Unit comprises PAHO/World Health Organization (WHO) and the Instituto Nacional de Enfermedades Infecciosas (INEI)-ANLIS “Dr. Carlos G. Malbrán” (RRL), who coordinates both activities and communication among members (Fig. 1).

## Innovation for Communication and Training

PNLAC works to strengthen communication between countries within the network. An innovative tool, a web conferencing platform, has been used since August 2008, to facilitate meetings and trainings. Up to date, the platform has hosted 28 conferences with an average of 12 countries participating each time. Some topics discussed include: outbreak investigations in each country, international alerts and news related to the International Health Regulations (IHR), troubleshooting on PFGE issues, quality assurance (QA) program (certifications/proficiency tests), and projects and reports from PNInt. The conferences are recorded and a summary is sent to all members. Training activities were also performed over the virtual platform, including: a complete advance course in PFGE BioNumerics (BN) analysis (2010: four sessions); a step-by-step video about PFGE laboratory procedures (2010: distributed to PNLAC and PNInt participants and uploaded to internet for general public access. http://www.youtube.com/user/pulsenetint?feature=watch); a workshop to enhance the knowledge on WGS with different international speakers (2017: seven sessions); and the organization of the annual meetings and workshops with the contribution of different countries. In recognition of PNLAC's of the advances done in the network and in new technologies for better communication and training for their members, PNLAC was awarded with the *IHRC Innovations in PulseNet* Award carried out in the 2013 Integrated Foodborne Outbreak Response and Management (InFORM) Conference on “PulseNet, OutbreakNet, and Environmental Health.” Additionally, INCIENSA, Costa Rica, an active member of PNLAC, was awarded for their achievements in introducing WGS during InFORM Association of Public Health Laboratories Conference in 2017.

Continuous training through courses and workshops were essential to keep the network updated. Over 14 seminar sessions, workshops, and training courses were performed during PNLAC Annual Meetings. Systematic training supported staff in improving management and interpretation of results to improve national and regional surveillance systems. Additional workshops were organized in collaboration with WHO to complement training with epidemiological topics related to foodborne illness source attribution, burden of diseases, and integrated surveillance for foodborne pathogens. Moreover, partnerships with different institutions worldwide were the primer to promote the initiative in the network for working in new technologies. As an example, in 2010, Center for Disease Control and Prevention (CDC) experts trained PNLAC members from Chile, Brazil, and Argentina in Multiple-Locus Variable-Number Tandem Repeat Analysis as a new strategy for molecular surveillance. In 2011, PNLAC worked to introduce WGS, and has continued providing trainings on WGS laboratory protocol and different strategies for sequence analysis.

Since 2005, a total of 16 PFGE training missions were important to strengthen the capacity in 13 countries and the Caribbean Region. Moreover, the RRL and other laboratories of the network have received visits from PNLAC members to reinforce the knowledge and abilities for PFGE implementation in their countries.

PNLAC network promotes the possibility of consulting with other members of the network or of other regional PulseNet networks or PNInt to work in cooperation to solve epidemiological issues. The exchange of experiences with other regions of PNInt is always an opportunity of learning. As example, PNLAC participated in the Asia Pacific (2010) and the Middle East (2010/2012) PulseNet Regional Meetings. As well as, in 2015, PNLAC had the opportunity to host members from other PulseNet regions in the framework of the Annual Meeting with PNInt.

## Strength of the Network

### QA/quality control

Up to date, the countries accomplish the QA/quality control (QC) program that supports the quality of the PFGE data, through the certification process of the organism(s) of interest for national surveillance; the certification is maintained by the Annual Proficiency Testing performed by both the RRL and CDC.

### Surveillance and outbreak investigation by PFGE

The technical and operational capacity of the network has improved gradually in each country according to the different social, economic, and cultural realities. At the beginning, PFGE implementation was incorporated in the surveillance strategies for *Salmonella* spp. and *Escherichia coli*. The protocol for the surveillance of *Shigella sonnei*, *Shigella flexneri*, *Listeria monocytogenes*, *Vibrio cholerae*, and *Vibrio parahaemolyticus* were adopted as requirement of the country.

In 2006, the RDB was created. It was a great effort for the region and different considerations were taken into account to have the installed capacity available for the participants. The server has been placed in PAHO, as a neutral location, and also to ensure the safe access from each country to the server, as well as the internet connectivity, security, and privacy. The maintenance and support of the RDB are performed by an IT staff member, trained at CDC for informatics and bioinformatics maintenance regional curators.

**FIG. 2.**
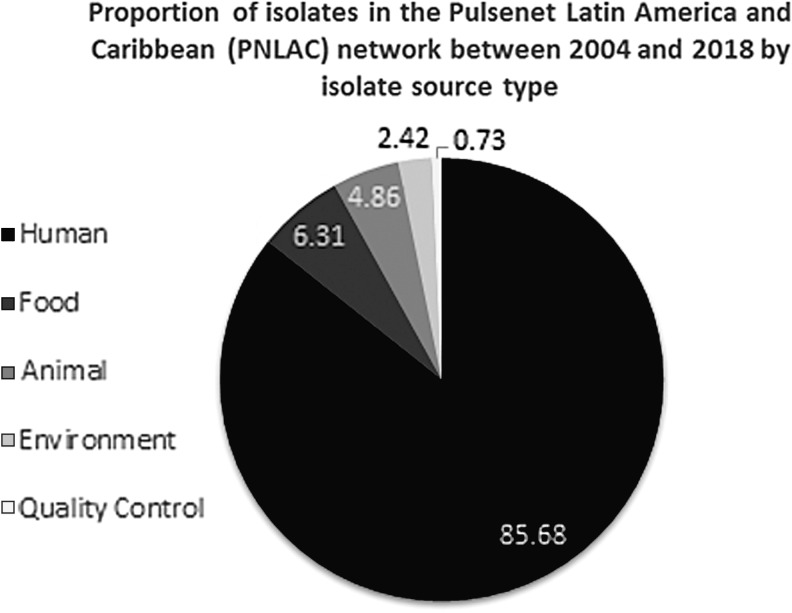
Proportional distribution of the isolates in the Regional Database of PulseNet Latin America and the Caribbean Network Regional Database by source of origin, from 2004 to 2017.

The RDB has increased in number with data from surveillance and different projects. Currently, PNLAC has the RDB for *Salmonella*, *Shigella*, *E. coli*, *L. monocytogenes*, *V. cholerae*, and *Campylobacter*. Among the 7602 isolates in the LAC PulseNet Database from 2004 to 2017, majority (85.7%) were isolated from humans, followed by food (6.3%) and animal (4.9%). Environmental and QA samples represented the lowest proportions of the isolates in the database (Fig. 2).

The data at RDB are complemented by those in each NDB, and through a consultation by email we could rapidly get the national information. With the RDB and NDB, the QC program, and the participants trained, each country could address outbreaks or clusters to give response to public health issues (Table 1).

**Table 1.** Outbreaks Studied by Pulsed-Field Gel Electrophoresis in the Last 2 Years in Countries of PulseNet Latin America and Caribbean

	*No. of outbreaks analyzed by PFGE*	*Countries*	*Pathogen associated*
*Salmonella*	88	Brazil, Guatemala, Paraguay, Perú, Uruguay, Colombia, Argentina	Serovar: Anatum, Derby, I, 4,5,12:i:-, Oranienburg, Infantis, Typhi, Enteritidis, Newport, Agona, Cerro, Montevideo, Javiana, Schwarzengrund, Worthintong, Bellevue, Uganda, Weltevreden, Hadar, Dublin, Braenderup, Lagos, Paratyphi, Gallinarum, Chester, London
*Shigella*	10	Colombia, Costa Rica, Argentina	*Shigella sonnei* (5), *Shigella flexneri* (5)
STEC	40	Argentina	*Escherichia coli* O157, *Escherichia coli* O121, *Escherichia coli* O145
*Vibrio*	1	Colombia	*Vibrio cholerae*
*Serratia*	2	Costa Rica	*Serratia liquefaciens*, *Serratia marcescens*
*Cronobacter sakazakii*	2	Costa Rica, Argentina	*C. sakazakii*
*E. coli*	1	Cuba	*DEC*

PFGE, pulsed-field gel electrophoresis; STEC, Shiga toxin-producing Escherichia coli.

During these years, the time from receiving the sample and uploading PFGE pattern to RDB has improved. We calculated time (number of days) from the date the isolate was received at the national laboratory to the date the PFGE pattern was uploaded to the RDB with the corresponding metadata between 2004 and 2018. The overall median time from the date the isolate was received at the laboratory to the date the isolate PFGE pattern was uploaded to the RDB was 187 d (range 1–1111 d), with marked reductions in time—approximately 48 d—between each progressive year the isolates were received since the network was set up.

The relationship and distribution of genetic subtypes of the pathogens monitored by the network, from the analysis of PNLAC RDB are presented below.

**FIG. 3.**
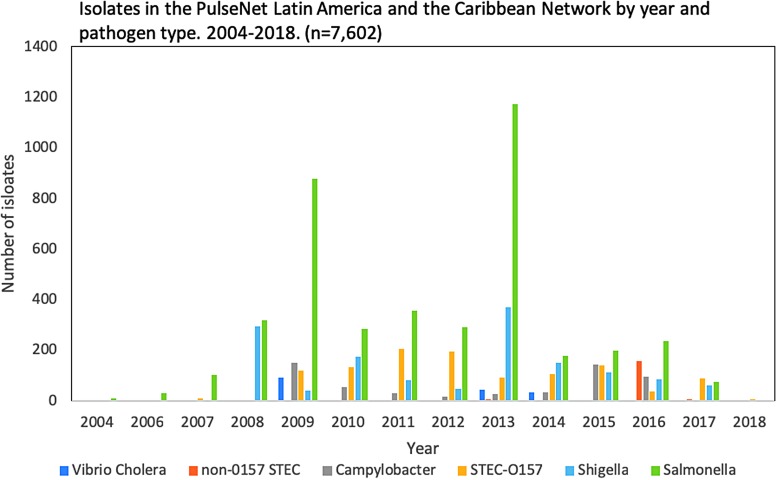
Isolates in the Regional Database of PulseNet Latin America and the Caribbean Network by year and pathogen type, from 2004 to 2018 (*n* = 7602). STEC, Shiga toxin-producing Escherichia coli.

Approximately 54.4% of the isolates received between 2004 and 2018 have been *Salmonella* spp., the most frequently reported pathogen to the RDB (Fig. 3).

#### *Salmonella enterica* serovar Typhi

*Salmonella enterica* serovar Typhi isolates were diverse, showing 329 *Xba*I-PFGE patterns among 967 isolates from 11 countries: Argentina, Bolivia, Brazil, Chile, Colombia, Costa Rica, Guatemala, Paraguay, Peru, Uruguay, and Venezuela. Of those, 43 patterns were found in more than one country. Of these shared patterns, ALJPPX01.0147 and ALJPPX01.0027 were associated with outbreak. Table 2, summarizes the information of ten most frequently identified *Xba*I-PFGE *Salmonella* Typhi patterns found in more than one country. The frequency of each pattern in the RDB, the country, and year of pattern upload are also shown. In 2012, an initial analysis was done with 48 *Salmonella enterica* serovar Typhi *Xba*I-PFGE patterns among 70 isolates from four countries between 2005 and 2009 (Campos *et al.*, 2012). None of those PFGE profiles was found in more than one country. In 2016, it was reported that 81% were isolated from sporadic cases and 19% from cases associated to outbreak. Only 6.79% of the strains showed antimicrobial resistance and 0.36% were multiresistant (Díaz *et al.*, 2016).

**Table 2.** *Salmonella enterica* Serovar Typhi XbaI Pulsed-Field Gel Electrophoresis Patterns Present in More Than One Country and Frequency of Distribution in the Regional Database from 2004 to 2017

*PFGE pattern no.*	*Frequency in data among* Salmonella *Typhi isolates (%)*	*Countries*	*Years*
ALJPPX01.0016	5.17%	Argentina	2004 and 2009
		Chile	2009, 2011 and 2013
		Colombia	2009, 2011 and 2013–2014
		Peru	2014
ALJPPX01.0048	3.93%	Argentina	2009
		Chile	2009 and 2013
		Colombia	2011 and 2013
ALJPPX01.0167	3.93%	Brazil	2013
		Chile	2013
ALJPPX01.0026	2.38%	Argentina	2009
		Brazil	2009
		Guatemala	2013–2014
ALJPPX01.0076	1.65%	Colombia	2009 and 2013
		Venezuela	2014
ALJPPX01.0080	1.45%	Brazil	2010
		Chile	2013
		Colombia	2009, 2011 and 2013
		Peru	2014
		Venezuela	2014
ALJPPX01.0123	1.34%	Chile	2013
		Colombia	2009, 2011 and 2013–2014
ALJPPX01.0147	1.24%	Chile	2011
		Colombia	2013
		Venezuela	2014
ALJPPX01.0019	1.03%	Argentina	2009
		Chile	2011 and 2013
		Colombia	2013
		Peru	2014
ALJPPX01.0027	1.03%	Argentina	2004
		Bolivia	2011
		Brazil	2013
		Chile	2013
		Colombia	2013
		Venezuela	2014

#### *Salmonella enterica* serovar Typhimurium

A total of 424 *Xba*I-PFGE patterns were identified in the RDB among 1322 *Salmonella enterica* serovar Typhimurium isolates from nine countries: Argentina, Barbados, Bolivia, Brazil, Chile, Colombia, Costa Rica, Guatemala, and Paraguay. Table 3, summarizes the information of the ten most frequent *Salmonella enterica* serovar Typhimurium *Xba*I-PFGE patterns found in more than one country. In 2012, it was reported that 226 *Xba*I-PFGE patterns were identified in the RDB among 554 *Salmonella enterica* serovar Typhimurium isolates from six countries: Argentina, Brazil, Chile, Colombia, Costa Rica, Paraguay (2005–2009) (Campos *et al.*, 2012).

**Table 3.** *Salmonella enterica* Serovar Typhimurium XbaI Pulsed-Field Gel Electrophoresis Patterns Present in More Than One Country and Frequency of Distribution in Database from 2004 to 2017

*PFGE-XbaI-pattern*	*Frequency*	*Countries*	*Years*
ALJPXX01.0027	7.34%	Argentina	2006–2016
		Brazil	2009
		Paraguay	2009
ALJPXX01.0054	2.34%	Argentina	2008–2013
		Brazil	2009
ALJPXX01.0068	1.21%	Argentina	2006 and 2008
		Brazil	2013
ALJPXX01.0094	1.13%	Argentina	2008–2017
		Chile	2010
ALJPXX01.0119	1.59%	Argentina	2010–2017
		Paraguay	2016
ALJPXX01.0154	4.61%	Argentina	2010–2016
		Brazil	2010, 2013 and 2015
ALJPXX01.0165	1.74%	Argentina	2009–2016
		Brazil	2010 and 2013
		Paraguay	2015–2016
ALJPXX01.0230	1.44%	Argentina	2010–2015
		Paraguay	2015
ALJPXX01.0267	1.21%	Argentina	2011–2016
		Paraguay	2016
ALJPXX01.0281	1.44%	Argentina	2010–2016
		Brazil	2010
		Paraguay	2016

#### *Salmonella enterica* serovar Enteritidis

A total of 41 *Xba*I-PFGE patterns were identified among 541 isolates from seven countries: Argentina, Bolivia, Brazil, Chile, Colombia, Guatemala, and Paraguay (Table 4). The predominant subtype that was identified in 2012—ALJEGX01.0001—that grouped 188 (83.5%) of the isolates, was once again determined to be the predominant subtype in this analysis as well (Campos *et al.*, 2012). *Salmonella enterica* serovar Enteritidis grouped 70.4% of the isolates reported from Argentina, Bolivia, Brazil, Chile, Colombia, Guatemala, and Paraguay over a span of 10 years (Table 4).

**Table 4.** *Salmonella enterica* Serovar Enteritidis XbaI Pulsed-Field Gel Electrophoresis Patterns Present in More Than One Country and Frequency of Distribution in Database from 2004 to 2017

*PFGE-XbaI-pattern no.*	*Frequency*	*Countries*	*Year*
ALJEGX01.0001	70.43%	Argentina	2006–2016
		Bolivia	2011 and 2013
		Brazil	2009–2015
		Chile	2009 and 2011
		Colombia	2009 and 2011
		Guatemala	2013
		Paraguay	2008–2009 and 2013
ALJEGX01.0010	8.32%	Argentina	2010, 2013, 2015, and 2016
		Brazil	2013
		Guatemala	2012
		Paraguay	2011 and 2013
ALJEGX01.0006	2.59%	Argentina	2007 and 2010
		Brazil	2013
		Colombia	2009 and 2011
ALJEGX01.0009	2.03%	Argentina	2007
		Brazil	2012, 2013 and 2016
ALJEGX01.0026	1.66%	Argentina	2016
		Paraguay	2011 and 2013
ALJEGX01.0004	1.48%	Argentina	2006–2007 and 2013
		Bolivia	2009
		Brazil	2013
ALJEGX01.0008	0.74%	Argentina	2007
		Brazil	2013
ALJEGX01.0035	0.55%	Argentina	2016
		Brazil	2013
ALJEGX01.0015	0.92%	Argentina	2006–2007 and 2012
		Colombia	2009

#### *Escherichia coli* O157:H7 and Shiga toxin producing Escherichia coli non-O157

There are 622 PFGE *Xba*I-PFGE patterns, corresponding to 1185 STEC O157 strains isolated in five countries of the region (Argentina, Chile, Cuba, Paraguay, and Uruguay), in the 1988–2015 period. There is a high clonal diversity, observing the circulation of characteristic subtypes in each of the countries. However, identical subtypes have been found in Argentina and Chile.

According to the information gathered in 2016 (Chinen *et al.*, 2016), data obtained from the surveillance systems for STEC in Argentina and Chile are presented in Table 5. The other countries showed differences in the frequency of diseases associated with STEC (Table 6).

**Table 5.** STEC Infection Cases in Argentina and Chile, with Consolidated Surveillance Systems

	*Chile 2007–2013*	*Argentina 2011–2015*
Samples received at NRL	2425	2119
Number of STEC strains	599 (24.7%)	587 (27.7%)
Most frequent serotypes	O157:H7, O26:H11 and O26:H-	O157:H7, O145:HNM and O121:H19
Age group	67.5% from 1 to 4 years old	80.5% from 1 to 4 years old
Gender	52.2% male/47.8% female	45% male/55% female

NRL, National Reference Laboratory.

**Table 6.** Latin America Countries with Low Frequency of STEC Infection Cases

*Country*	*STEC associated cases (diarrhea/HUS)*	*STEC strain isolated*
Bolivia	2 HUS	O157 *stx*_2_; O26 *stx*_1_
Brazil	41 diarrhea	no-O157
Costa Rica	11 diarrhea	O157 *stx*_2_ (2); O145 *stx*_1_ (3); ONT *stx*_1_ (2); ONT *stx*_1_*eae* (4)
Cuba	4 diarrhea	no-O157 stx_2_ eae
Paraguay	7 diarrhea	no-O157: *stx*_1_; *stx*_1_*eae*; *stx*_1_/*stx*_2_*eae*; *stx*_2_ (3); O26 *stx*_1_/*stx*_2_
Perú	4 diarrhea	O157:H7: *stx*_2_ (2); *stx*_1_; *stx*_1_/*stx*_2_

HUS, Haemolytic uraemic syndrome.

The most frequent serotypes in the Region are O157:H7 (56.3%), O145:H- [fliCH28], and O26:H11/H- (13.4%). In general, cases of Haemolytic uraemic syndrome (HUS) are sporadic, however, outbreaks were reported mainly in a family or in the community.

STEC surveillance represents a challenge in the region due to the severity and sequelae of the diseases it produces, the emergence of new pathotypes and the ability to produce outbreaks. Detection of STEC O157 was associated with two cases of urinary infection in adult women of 72 and 84 years, in Uruguay, in 2010 (Gadea *et al.*, 2012). Detection of 19 hybrid strains EAEC/STEC O59: NM [fliCH19], was associated with cases of HUS and bloody diarrhea, in Argentina, 2005–2015 (Carbonari *et al.*, 2018).

In LA, the notification of diseases associated with STEC and cases of HUS is mainly carried out through syndromic surveillance, through the surveillance of foodborne diseases (Bolivia, Cuba and Paraguay), and/or through the surveillance system of acute diarrhea (Argentina, Brazil, Chile, Costa Rica, Paraguay, and Peru). The notification of HUS cases is mandatory in Argentina, Bolivia, Brazil, Chile, and Paraguay. Uruguay and Costa Rica do not have a formal surveillance system for HUS and STEC infections. In general, syndromic surveillance is reinforced with laboratory-based surveillance through its National. The HUS is endemic in some countries of the Southern Cone, being Argentina the country with the highest incidence worldwide, ∼10 cases/100,000 children younger than 5 years of age in 2015.

#### *Campylobacter* spp

The RDB has 533 *Campylobacter* spp. isolates corresponding to 249 PFGE patterns (470 *C. jejuni* strains—206 *Xba*IPFGE-patterns; 63 *C. coli* strains—43 *Xba*IPFGE-patterns). They were isolated in Argentina (*n* = 514) and Paraguay (*n* = 19) (Table 7).

**Table 7.** Latin America Countries with *Campylobacter jejuni* Infection Cases

*PFGE pattern no.*	*Frequency in data among* C. jejuni *isolates (%)*	*Countries (isolates)*	*Years*
ALDBRS16.0001	2.81%	Argentina (15)	2009, 2011, 2015 and 2016
ALDBRS16.0005	3.00%	Argentina (15) and Paraguay (1)	2007, 2009, 2010, 2013, 2014, 2015 and 2016
ALDBRS16.0034	6.94%	Argentina (36) and Paraguay (1)	2006, 2007, 2008, 2009, 2010, 2011, 2012, 2014, 2015 and 2016
ALDBRS16.0051	3.00%	Argentina (14) and Paraguay (2)	2006, 2007, 2009, 2013, 2014, 2015 and 2016
ALDBRS16.0105	1.88%	Argentina (10)	2009, 2011, 2013, 2014, 2015 and 2016

The *C. jejuni* and *C. coli* strains isolated in Argentina and Paraguay, have shown diversity by *Sma*I-PFGE. It was interesting to detect the same pattern in both countries suggesting the importance of the investigation in food, as infection source and vehicle of dissemination.

#### Listeria monocytogenes

The RDB has 208 *L. monocytogenes* isolates from human and food, corresponding to 66 *Apa*I-PFGE patterns. They were isolated in Argentina (*n* = 514; human), Costa Rica (*n* = 47; human and food), and Chile (*n* = 18; food and human) (Table 8).

**Table 8.** Latin America Countries with *Listeria monocytogenes* Infection Cases

*PFGE pattern no.*	*Frequency in data among* L. monocytogenes *(%)*	*Countries/origin (isolates)*	*Years*
ALGX6A12.0001	3.8%	Argentina/human (8)	1993, 1995, 1998 and 2006
ALGX6A12.0005	3,8%	Argentina/human (8)	1999, 2000, 2001, 2002 and 2007
ALGX6A12.0006	7.2%	Argentina/human (15)	2011, 2012, 2013 and 2015
ALGX6A12.0008	8.6%	Argentina/human (18)	2011 and 2013
ALGX6A12.0047	1.9%	Costa Rica/human, food (4)	—
ALGX6A12.0039	2.4%	Costa Rica/food (5)	2008 and 2013
ALGX6A12.0057	2.8%	Costa Rica/food (6)	—

#### Shigella sonnei

The RDB includes 1283 *S. sonnei* strains, isolated from Argentina (*n* = 978), Colombia (*n* = 101), Costa Rica (*n* = 52), Paraguay (*n* = 51), Brazil (*n* = 28), Chile (*n* = 24), and Perú (*n* = 49). Out of the total 1273 were analyzed by *Xba*I (633 different *Xba*I-PFGE patterns) and 109 by *Bln*I (57 different *Bln*I-PFGE patterns).

#### Vibrio cholerae

There are data from 172 isolates at the *V. cholerae* RDB from 1992 to 2018 from Argentina, Brazil, Chile, Colombia, Ecuador, Paraguay, and Trinidad and Tobago. Out of which, 134 were analyzed by *Sfi*I-PFGE and 74 by *Not*I-PFGE.

The RDB was useful to study the outbreak (*n* = 356) that occurred in Venezuela (López, 2011) with imported cases from República Dominicana on January 22, 2011. By *Sfi*I-PFGE the strains showed a 98% of similarity with one of the Haiti strains, but were different by *Not*I-PFGE. The outbreak was notified to the focal point of IHR, as a public health event of international concern.

### Methodological contributions

PulseNet laboratories worldwide use PFGE standardized protocols (CDC, Atlanta, GA) for the surveillance of the main pathogens and the participants apply them according to their priorities in each country. In the light of the need in LA to have specific PFGE procedures for *Cronobacter sakazakii* and *S. flexneri*, PNLAC had developed and validated their protocols in contribution to improve the laboratory-based surveillance of these pathogens worldwide.

In 2005–2008 in Argentina, *C. sakazakii* was detected from commercial brands of imported powdered infant formula and not from any human cases until that moment. To have a specific PFGE protocol for subtyping Cronobacter species, an initial development and multicenter validation process (Brengi *et al.*, 2012) was done at INEI-ANLIS, Argentina, working together with four other laboratories: CDC, United States; the National Microbiology Laboratory of Health Canada, Canada; the World Health Organization (WHO) Cronobacter Reference Laboratory, University College Dublin, Ireland; and the Institute for Food Safety and Hygiene, University of Zurich, Switzerland. This protocol was useful to investigate the first episode of fatal cases of neonatal infections associated with *Cronobacter malonaticus* and *C. sakazakii* in the same hospital in Argentina that occurred from January 2009 through September 2010 (Asato *et al.*, 2013). Also another fatal case associated with *C. sakazakii* occurred in Costa Rica was studied and it demonstrated that the same strain was isolated from the girl and the powdered infant formula that she had sampled at home. The investigation demonstrated that it was related to improper handling of the product.

*S. flexneri* is one of the species more prevalent in LA as well as in less developed regions. Additionally, *S. flexneri* is the most frequently species isolated worldwide; and often related as the cause of outbreaks in settings with poor hygiene or sanitary conditions, and imported cases into developed countries associated with travel. With the objective of promoting the surveillance of *S. flexneri*, a new protocol (Pichel *et al.*, 2012), highly robust and reproducible, was proposed for subtyping *S. flexneri*. A multicenter study was conducted in nine PulseNet laboratories located in North and South America, Europe, and Asia [INEI-ANLIS “Dr Carlos G. Malbrán” (INEI-ANLIS) of Argentina (co-coordinating laboratory), U.S. Centers for Disease Control and Prevention (CDC; co-coordinating laboratory), Instituto Adolfo Lutz of Brazil, National Microbiology Laboratory of Canada, Instituto de Salud Pública de Chile, Statens Serum Institute of Denmark, Global Disease Detection (GDD) Regional Center at the U.S. Naval Medical Research Unit 3 (NAMRU-3), Egypt, Central Public Health Laboratory of Oman, and Public Health Laboratory Center of Hong Kong, China].

### PNLAC moving to WGS

As one of the aims of PNLAC of improved laboratory-based surveillance according to the international standards, WGS has been established as a new tool in the network, starting as a complement for the existing techniques and according to the national capacities of each network members, giving support to the ones that has no access to the technology, as it started with PFGE.

The implementation of PNLAC started in 2011 in the frame of a regional project of *S. sonnei* to study the diversity in the region and in a global context, and to evaluate the use of WGS for the network for laboratory-based surveillance, including outbreak detection (Baker *et al.*, 2017), with the Wellcome Trust Sanger Institute (WTSI) as partner.

At the beginning, capacity building in genome analysis was the priority, organizing different courses with different analysis approaches, including single nucleotide polymorphisms, and the gene-by-gene based—that is, extended multilocus sequence typing based on WGS.

With sequencing capacity increasing in the region since 2014, the wet laboratory training was incorporated in the courses and the participants of the networks were trained in different workshops and during PNLAC Annual Meetings.

International partners were the key for adopting this new technology in the region for foodborne surveillance, including CDC, Food and Drug Administration, WHO, and WTSI.

In 2016, a working group, including Argentina, Chile, Colombia, and México (Perú was incorporated in 2018) was created to establish future steps for using WGS in PNLAC.

In 2018, most of the PNLAC members (México, Perú, Venezuela, Chile, Argentina, Panamá, Paraguay, Costa Rica, Uruguay, Colombia, and Brazil) had local sequencing capacity (Illumina technology, except Brazil that has Ion Torrent).

The working group agreed in a regional analysis workflow and agreed in the creation of a Regional WGS Database with the principles of the PFGE database in data handling and privacy policies.

In 2018, the working group agreed in a wet laboratory protocol, including optional steps for the variations at the national level, and is working in a regional QC program for WGS and a regional pipeline with regional IT capacity to support the countries. For the countries who need support for the bioinformatics analysis in the first stage of genomic implementation, the working group provides the genomic analysis for specific surveillance questions using the agreed workflow for the network.

## Conclusions

In 2018, PNLAC celebrated its 15th anniversary as a consolidated laboratory-based surveillance Regional Network in foodborne and other enteric pathogens with 24 laboratory members. The network has been installing the capacity, including infrastructure, expert personnel in PFGE, and is moving toward WGS to continue their contribution to enhance the National and Regional Public Health System, in agreement with international standards.
